# Quantification in cardiovascular magnetic resonance: agreement of software from three different vendors on assessment of left ventricular function, 2D flow and parametric mapping

**DOI:** 10.1186/s12968-019-0522-y

**Published:** 2019-02-21

**Authors:** Leonora Zange, Fabian Muehlberg, Edyta Blaszczyk, Susanne Schwenke, Julius Traber, Stephanie Funk, Jeanette Schulz-Menger

**Affiliations:** 1 Working Group on Cardiovascular Magnetic Resonance, Experimental and Clinical Research Center, a joint cooperation between the Charité Medical Faculty and the Max-Delbrueck Center for Molecular Medicine and HELIOS Hospital Berlin-Buch, Department of Cardiology and Nephrology, Medical University Berlin, Charité Campus Buch, Lindenberger Weg 80, 13125 Berlin, Germany; 2 DZHK (German Centre for Cardiovascular Research), partner site Berlin, Berlin, Germany; 3 SCO:SSiS, Berlin, Germany; 4 Department of Cardiology and Angiology, Medizinische Klinik II, Klinikum Braunschweig gGmbH, Braunschweig, Germany

**Keywords:** Cardiovascular magnetic resonance, Analysis, Post-processing software, Left ventricle, 2D flow, Parametric mapping

## Abstract

**Background:**

Quantitative results of cardiovascular magnetic resonance (CMR) image analysis influence clinical decision making. Image analysis is performed based on dedicated software. The manufacturers provide different analysis tools whose algorithms are often unknown. The aim of this study was to evaluate the impact of software on quantification of left ventricular (LV) assessment, 2D flow measurement and T1- and T2-parametric mapping.

**Methods:**

Thirty-one data sets of patients who underwent a CMR Scan on 1.5 T were analyzed using three different software (Circle CVI: cvi^42^, Siemens Healthineers: Argus, Medis: Qmass/Qflow) by one reader blinded to former results. Cine steady state free precession short axis images were analyzed regarding LV ejection fraction (EF), end-systolic and end-diastolic volume (ESV, EDV) and LV mass. Phase-contrast magnetic resonance images were evaluated for forward stroke volume (SV) and peak velocity (Vmax). Pixel-wise generated native T1- and T2-maps were used to assess T1- and T2-time. Forty-five data sets were evaluated twice (15 per software) for intraobserver analysis. Equivalence was considered if the confidence interval of a paired assessment of two sofware was within a tolerance interval defined by ±1.96 highest standard deviation obtained by intraobserver analysis.

**Results:**

For each parameter, thirty data sets could be analyzed with all three software. All three software (A/B, A/C, B/C) were considered equivalent for LV EF, EDV, ESV, mass, 2D flow SV and T2-time. Differences between software were detected in flow measurement for Vmax and in parametric mapping for T1-time. For Vmax, equivalence was given between software A and C and for T1-time equivalence was given between software B and C.

**Conclusion:**

Software had no impact on quantitative results of LV assessment, T2-time and SV based on 2D flow. In contrast to that, Vmax and T1-time may be influenced by software. CMR reports should contain the name and version of the software applied for image analysis to avoid misinterpretation upon follow-up and research examinations.

**Trial registration:**

ISRCTN12210850. Registered 14 July 2017, retrospectively registered.

**Electronic supplementary material:**

The online version of this article (10.1186/s12968-019-0522-y) contains supplementary material, which is available to authorized users.

## Background

In the past years, cardiovascular magnetic resonance Imaging (CMR) has emerged as a broadly applied imaging modality in cardiac diagnostics [1]. Due to its high accuracy and reproducibility, CMR is considered as gold standard for evaluation of left ventricular (LV) function [2]. CMR is the recommended method to assess cardiac function and hemodynamics especially when transthoracic echocardiography is limited [3]. In addition to mere functional assessment, non-invasive tissue differentiation represents CMR’s unique feature [4]. Clinical decision-making is often based on quantification, i.e. the placement of an implantable cardiac defibrillator depends on quantified LV function or valve replacement on quantitative flow assessment [5–7]. Therefore, accurate and reliable quantification is essential for correct diagnosis and adequate treatment. Technical aspects such as field strength, vendor platforms and imaging protocol influence CMR results [8–12]. The Society for Cardiovascular Magnetic Resonance (SCMR) published not only standardized protocols for image acquisition and interpretation, but also guidelines for reporting which propose to report scanner type, sequences used and study quality [13–15]. Interestingly, it is not suggested to report the software used [15]. CMR image analysis is performed on dedicated commercial and non-commercial software solutions. They often differ within and between sites. Quantitative analysis is mostly based on manually contouring or manually correction of semi-automatic segmented regions of interest (ROI) in CMR images. For LV volumetry and flow quantification, the contour relies on the definition of a whole pixel or subpixel depending on the software. In case of parametric mapping, not all software providers do have a specific tool. A recent study reported that software used for myocardial perfusion analysis is not interchangeable and reliable results were only achieved within the same software [16]. In contrast to this, statistically significant differences were found in analysis of T2* mapping between two software which were considered to be without any effect on clinical decision making [17]. Other groups found a strong correlation and no significant differences between software for LV assessment [18, 19]. Software comparison for flow measurement was only done in a small number of patients [20]. The impact of the software-dependent approaches of contour modification on results is unknown and mathematical calculation and extrapolation remain reserved to the vendors.

The aim of the present study was to investigate the equivalence of three commercially available software used at our site for assessment of LV, 2D flow and T1- and T2-parametric mapping. We hypothesized that mean differences between software are smaller than intraobserver variability and hence, software can be considered as equivalent.

## Methods

### Patient data sets

For logistical reasons, we chose at the beginning of this study the first available data sets of patients with histologically confirmed soft tissue sarcoma planned for anthracycline-based chemotherapy from an on-going study of our working group (ISRCTN12210850) [21]. Exclusion criteria were chronic renal failure (estimated glomerular filtration rate < 30 mL/m^2^), cardiac metastases, known incompatibility for gadolinium contrast media and contraindication for CMR. We had to exclude short axis images (SAX) in one patient as they were not recorded continuously, T1-map in one patient due to an artifact and flow in one patient due to aliasing in flow measurement. In order to still match the required number of analyzed data, we included a 31st patient for analysis of SAX, T1-time and flow. A total of 31 data sets of patients (16 male, details see Table 1) were analyzed. The population suffered from different co-morbidities. In detail, 15 patients (48%) had arterial hypertension, 1 patient (3%) had coronary artery disease and 6 patients had diabetes mellitus type II (19%). Eleven patients (36%) received anthracycline-based chemotherapy (> 300 mg/m^2^ doxorubicin-equivalent cumulative dose) prior to the study. Ethical approval was given for the mentioned study by the local ethics committee of Charité Medical University Berlin (approval number EA1/262/14). All patients gave their written informed consent before participating in the study.

**Table 1:** Patient characteristics

Variables	Patients (*n* = 31)
Age [years]	60 ± 14
Height [cm]	172.7 ± 9.5
Weight [kg]	80.1 ± 18.9
Body surface area [m^2^]	1.95 ± 0.27
Systolic blood pressure [mmHg]	119.3 ± 17.4
Diastolic blood pressure [mmHg]	71.7 ± 10.9
Heart rate [bpm]	68.9 ± 18.1

Values are presented as mean ± standard deviation

### CMR imaging protocol

All CMR examinations were performed using a 1.5 Tesla scanner (Magnetom Avanto Fit, Siemens Healthineers, Erlangen, Germany). Protocol and slice planning were identical in all cases according to institutional standards. In short, retrospective electrocardiographic (ECG) gated balanced steady state free precession (bSSFP) cine images covering the whole LV from basis to apex were obtained without gap in a breath-hold technique (repetition time 46.34 ms, echo time 1.44 ms, voxel size 2.0 × 2.0 × 7.0 mm^3^, flip angle 80 degrees). Segmented gradient-echo phase contrast CMR (PC-CMR) was performed at the sinotubular junction of the ascending aorta. The velocity encoding range was set at 150 cm/s in a through-plane direction [9]. Native T1- and T2-mapping data were obtained in one midventricular short axis as previously described [4].

### Post-processing software packages

**Fig. 1:**
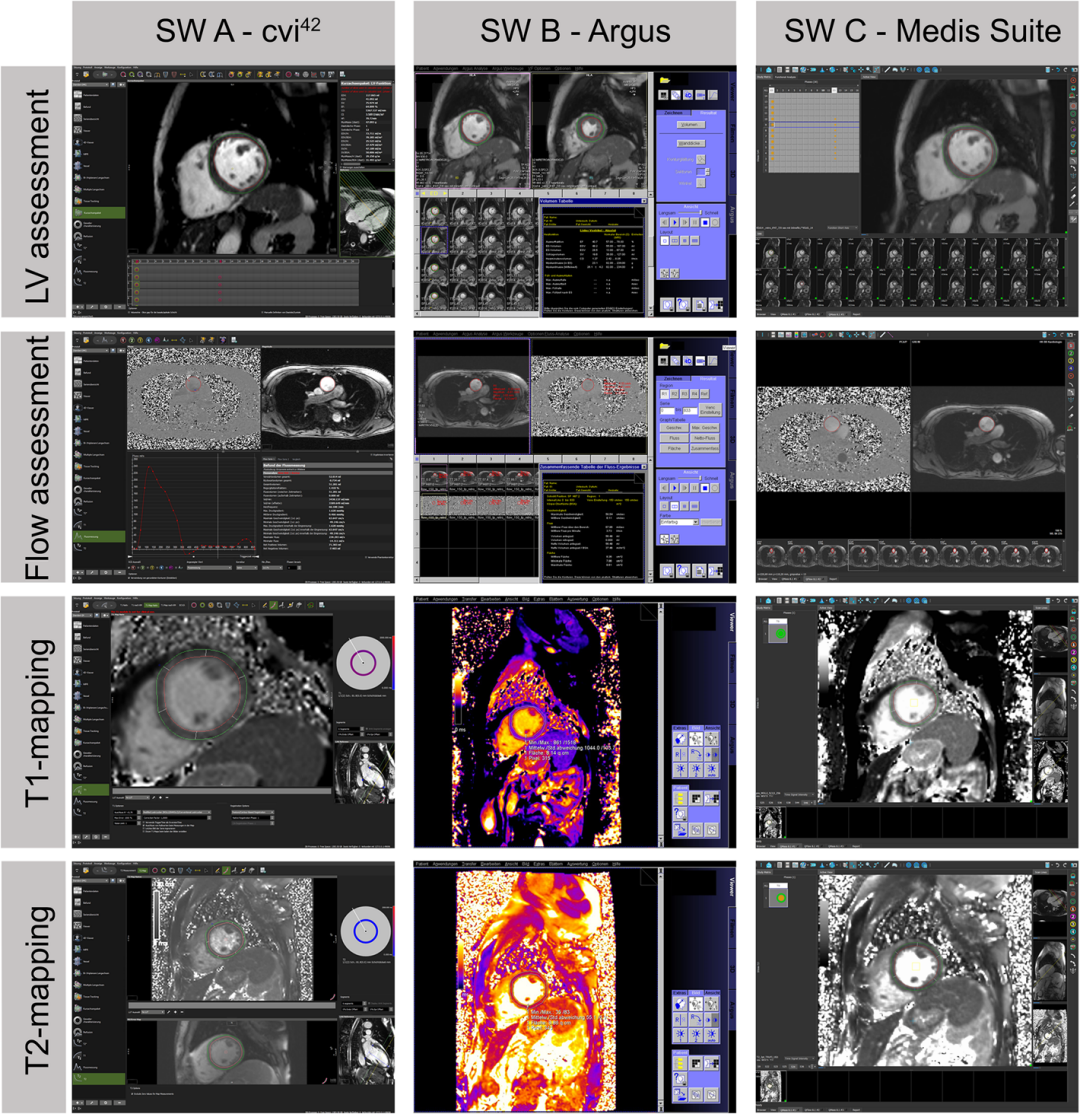
Presentation of software surfaces. Screenshots of cvi^42^, Argus and Medis Suite used for image analysis for left ventricular assessment, 2D flow measurement and T1- and T2-parametric mapping

Three software packages were used for image analysis and quantitative assessment according to current institutional standards between June and December 2016 [13]. All data sets were analyzed by one reader (L.Z.) blinded to former quantitative results using Circle Cardiovascular Imaging: cvi^42^ version 5.3.2 (Calgary, Canada), (software A); Siemens Healthineers: Classic Argus (Argus viewer, Argus LV function and Argus flow) on SyngoMMWP version VE53A acquisition work place, (software B); and Medis medical imaging systems: Medis Suite 2.1 with the applications Qmass and Qflow version 8.1 (Leiden, Netherlands), (software C). We analyzed software using the default settings. The software surfaces are presented in Fig. 1. Forty-five data sets randomly selected were analyzed twice (15 per software) for intraobserver analysis.

### Left ventricular assessment

For LV assessment we used cvi^42^ heart function [22], Argus LV function [23] and Qmass ventricular function [24]. Endo- and epicardial borders were contoured manually in short axis cine images (SAX) at end-diastole and end-systole. The basal slice was included in the analysis if at least 50% of blood volume was surrounded by myocardium. Papillary muscles were excluded and considered part of the blood pool. If available, contour smoothing was applied. Quality control of the contours was performed in the movie mode. Ejection fraction (EF in %) and myocardial mass (Mass in g), end-systolic and end-diastolic volume (ESV and EDV, respectively, in ml) were recorded [25].

### Flow measurement

For 2D flow assessment, we used cvi^42^ Flow [26], Argus Flow [27] and Qflow PC Flow [28]. The ascending aorta was contoured in the magnitude image with the sharpest blood/tissue contrast. Contours were propagated to phase contrast images in all temporal phases, corrected manually and controlled carefully. Peak velocity (Vmax in cm/s) was measured in all software and forward stroke volume (SV in ml) was calculated automatically in function of the vessel area in all phases [29].

### Parametric mapping

For T1 and T2 mapping the procedure was identical in cvi^42^ (using T1- and T2-tool) [30, 31] and Qmass (using time signal intensity mode) [32]. Endo- and epicardial limits were delineated and corrected in all 8 raw images for T1-mapping or 3 raw images for T2-mapping, copied into the scanner generated pixel maps and corrected again if necessary. In Argus viewer [33], a ROI was drawn around the myocardium in all colored pixel-wise maps with the same procedure for all studies. Segment-based global T1- and T2-times (in ms) and area (if available) were recorded [34, 35].

### Statistical analysis

The sample size calculation for the equivalence test was based on reference values obtained with cvi^42^ in our working group and from literature assuming that the distribution of the available data is comparable to other software types [9, 36, 37]. The equivalence margin was set to the 95% tolerance interval of the intraobserver difference with 95% coverage, such that two software systems would be considered equivalent if their deviations would be within the limits of 95% of the deviances generated by one observer performing repeated measures with the same software. It was assumed that the standard deviation (SD) of each software would be equal to the intraobserver variability. Based on a power of 0.9 and a Bonferroni-corrected α-level of 0.017 correcting for three tests, 30 patients were found to be sufficient even for the conservative assumption of a correlation of 0.2 between measurements of two different software. PASS, version 11, was used for sample size calculation [38].

Normality was checked based on visual inspection of the data using Quantile-Quantile-plots (QQ-plots). No strong deviations from normal distribution were noted thus parametric methods were used. The Pearson’s correlation coefficient (r) was calculated for correlation analysis and Bland-Altman plots were generated to assess the bias (mean difference) and the 95% limits of agreement between each pair of software for each parameter. Equivalence limits were determined as ±1.96 maximum intraobserver SD variability across the three software, which corresponds to the largest observed 95% tolerance interval with 95% coverage of repeated measurements with the same software. Following the approach outlined by Walker and Nowacki [39], Bonferroni corrected confidence intervals were constructed using α = 0.05/3 = 0.017, thus leading to (1–2α)*100% = 96.7% confidence intervals. These were obtained for the paired assessments of two software and equivalence was concluded when the confidence interval was completely within the limits of equivalence. Testing the null hypothesis of no difference between software was based on a test with shifted null hypothesis where the shift equaled the respective limits of equivalence. As the results of an equivalence test by CI is only binary (yes/no), no *p*-values were given. Area of T1- and T2-mapping was recorded if applicable and compared for differences by paired t-test with α = 0.05. Statistical analysis was performed by Graph Pad Prism 6, version 6.0.7 for windows [40].

## Results

**Fig. 2:**
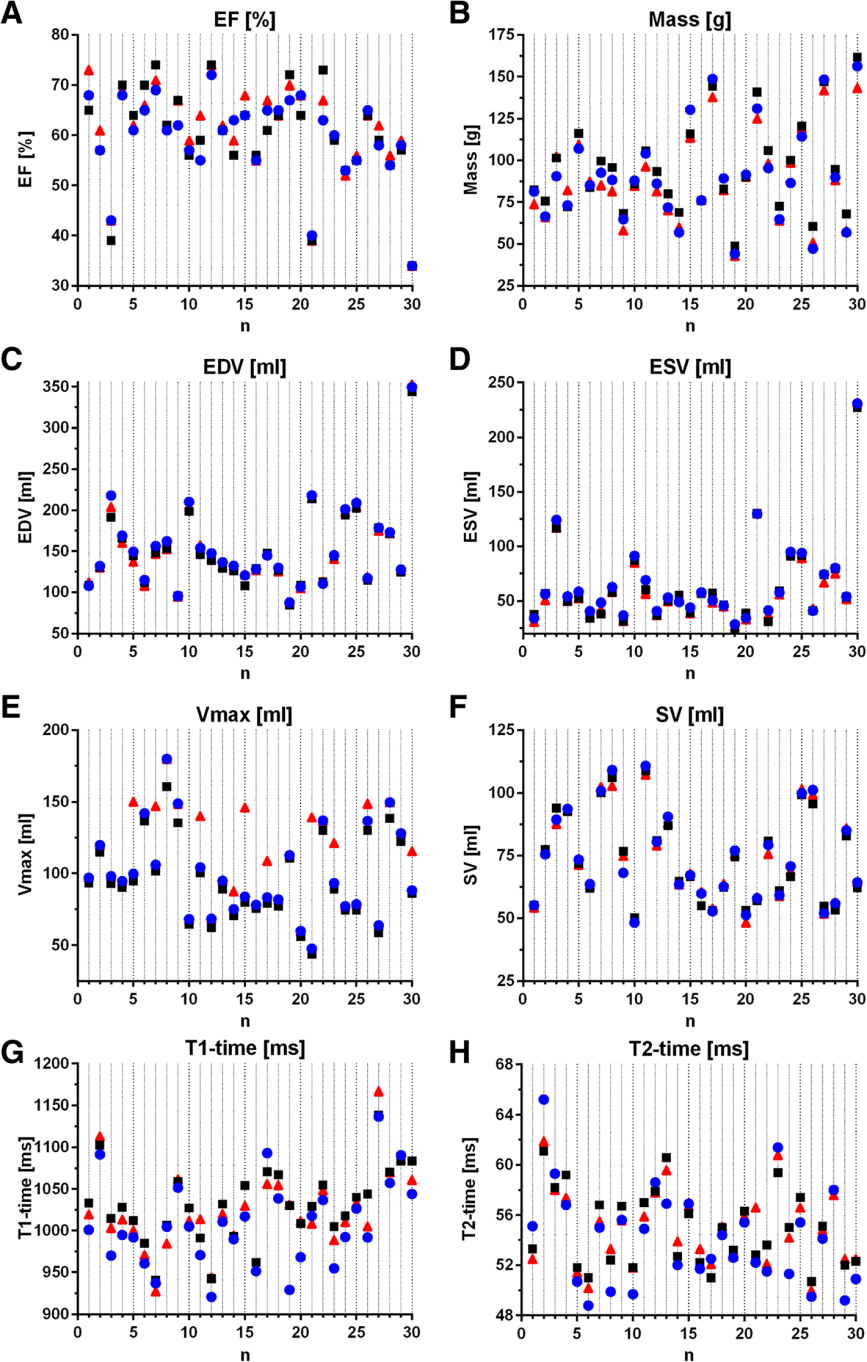
Single values obtained for each patient with each software for left ventricular assessment (**a**-**d**), 2D flow (**e**, **f**) and parametric mapping (**g**, **h**). EF: ejection fraction, EDV end-systolic volume, ESV end-diastolic volume, Vmax: peak velocity, SV: stroke volume. Blue dot: Software A; black square: Software B; red triangle: Software C

For each parameter 30 data sets were available and could be analyzed with each software (Fig. 2 and Table 2).

**Table 2:** Results of left ventricular assessment, 2D Flow and parametric mapping per software (A, B, C)

	Software A	Software B	Software C
Left ventricular assessment			
Ejection Fraction [%]	59.5 ± 8.6	60.1 ± 9.8	61.1 ± 9.4
Mass [g]	90.8 ± 29.1	95.2 ± 27.3	89.0 ± 26.5
End-diastolic volume [ml]	154.4 ± 51.7	149.6 ± 49.7	151.5 ± 51.1
End-systolic volume [ml]	65.8 ± 40.0	63.4 ± 39.8	62.6 ± 40.4
2D Flow measurement			
Vmax [cm/s]	98.0 ± 29.6	92.8 ± 27.4	98.0 ± 29.6
Stroke Volume [ml]	75.7 ± 19.2	75.3 ± 18.5	72.5 ± 18.4
Parametric mapping			
T1-time [ms]	1008.1 ± 51.9	1030.8 ± 44.5	1023.2 ± 48.8
T2-time [ms]	54.2 ± 3.9	55.0 ± 3.1	54.9 ± 3.0

Values are presented as mean ± standard deviation

*EF* ejection fraction, *EDV* end-systolic volume, *ESV* end-diastolic volume, *Vmax* peak velocity, *SV* stroke volume

### Left ventricular assessment

**Fig. 3:**
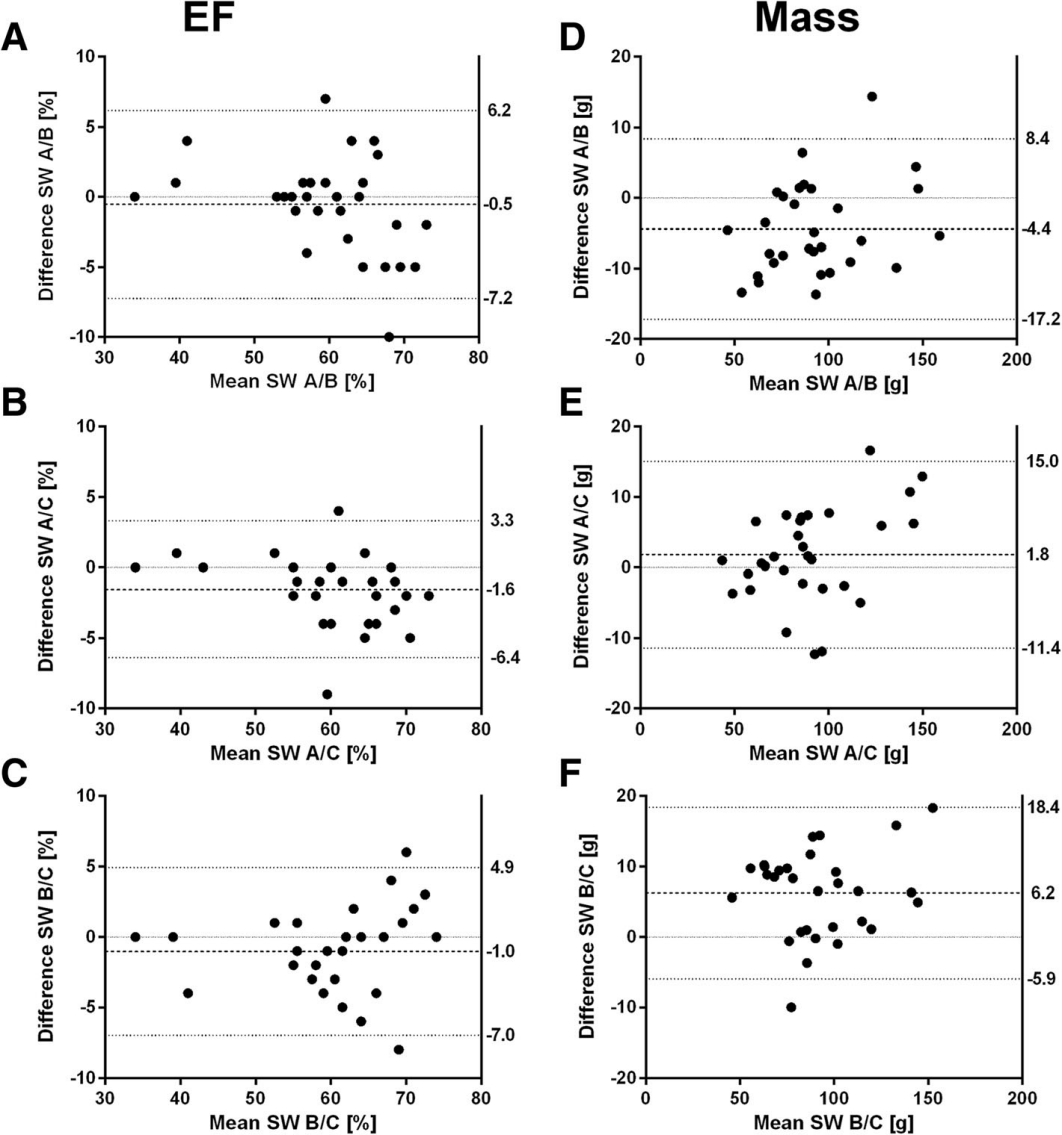
Bland-Altman plots of LV function (EF) and LV mass for agreement between software A and B (**a**, **d**), software A and C (**b**, **e**) and software B and C (**c**, **f**). Dashed lines indicate mean difference, dotted lines indicate limits of agreement

**Fig. 4:**
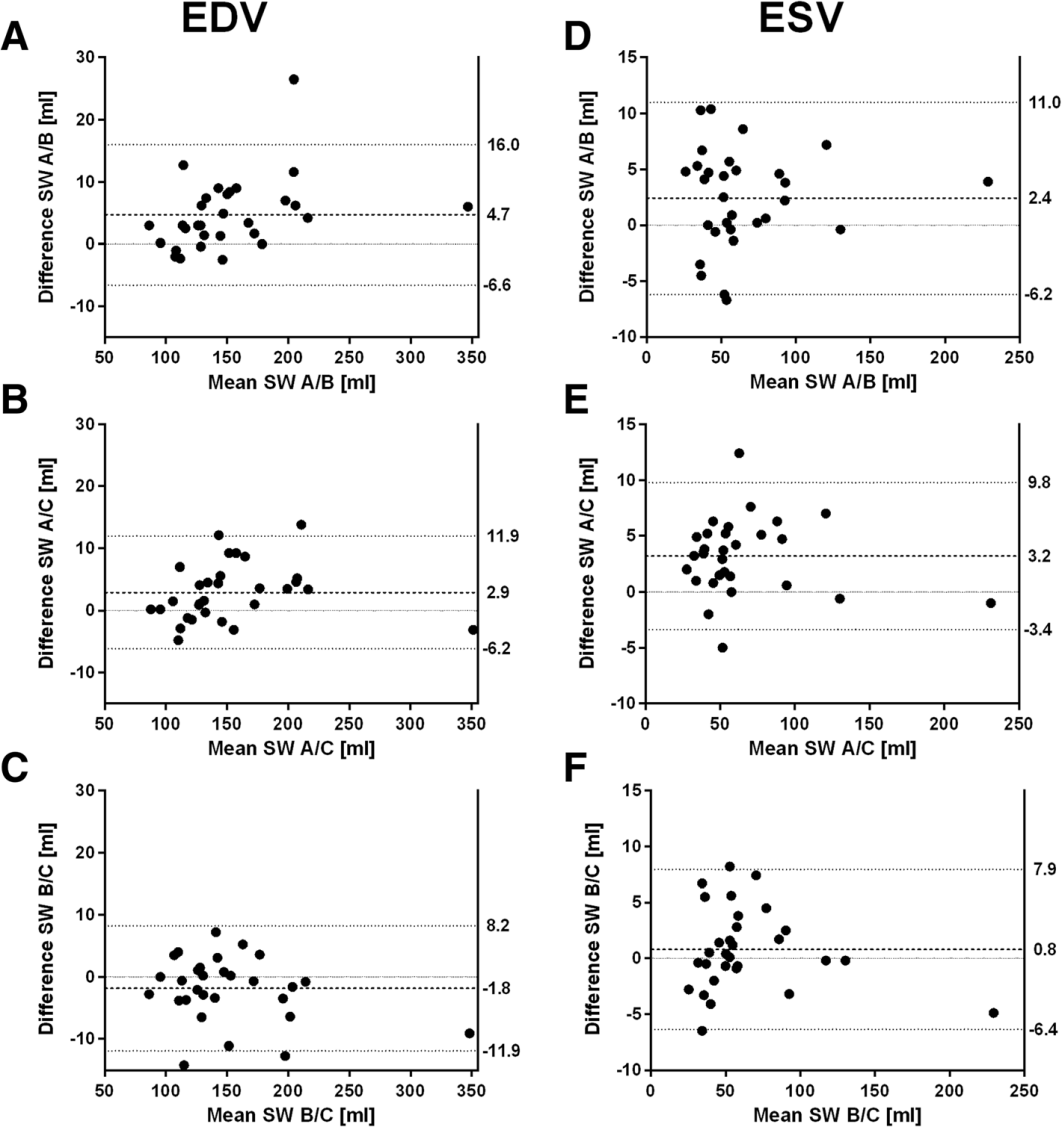
Bland-Altman plots of LV end-diastolic (EDV) and end-systolic volume (ESV) for agreement between software A and B (**a**, **d**), software A and C (**b**, **e**) and software B and C (**c**, **f**). Dashed lines indicate mean difference, dotted lines indicate limits of agreement

All software showed a strong positive correlation for EF (r software A/B: 0.940, software A/C: 0.965, software B/C: 0.951), mass (r software A/B: 0.975, software A/C: 0.975, software B/C: 0.974), EDV (r software A/B: 0.994, software A/C: 0.996, software B/C: 0.995) and ESV (r software A/B: 0.994, software A/C: 0.997, software B/C: 0.996). For EF, Bland-Altman analysis revealed narrowest limits of agreement between software A/C (Fig. 3b). Smallest bias but widest limits of agreement were found between software A/B (Fig. 3a). Comparing software B/C, a rising difference with increasing mean is shown (Fig. 3c). For mass, bias of software A/B was more than twice and B/C more than three times higher compared to software A/C (Fig. 3d-f). For EDV and ESV, narrowest limits of agreement were found between software A/C, but smallest bias was detected between software B/C (Fig. 4a-f).

### Flow measurement

**Fig. 5:**
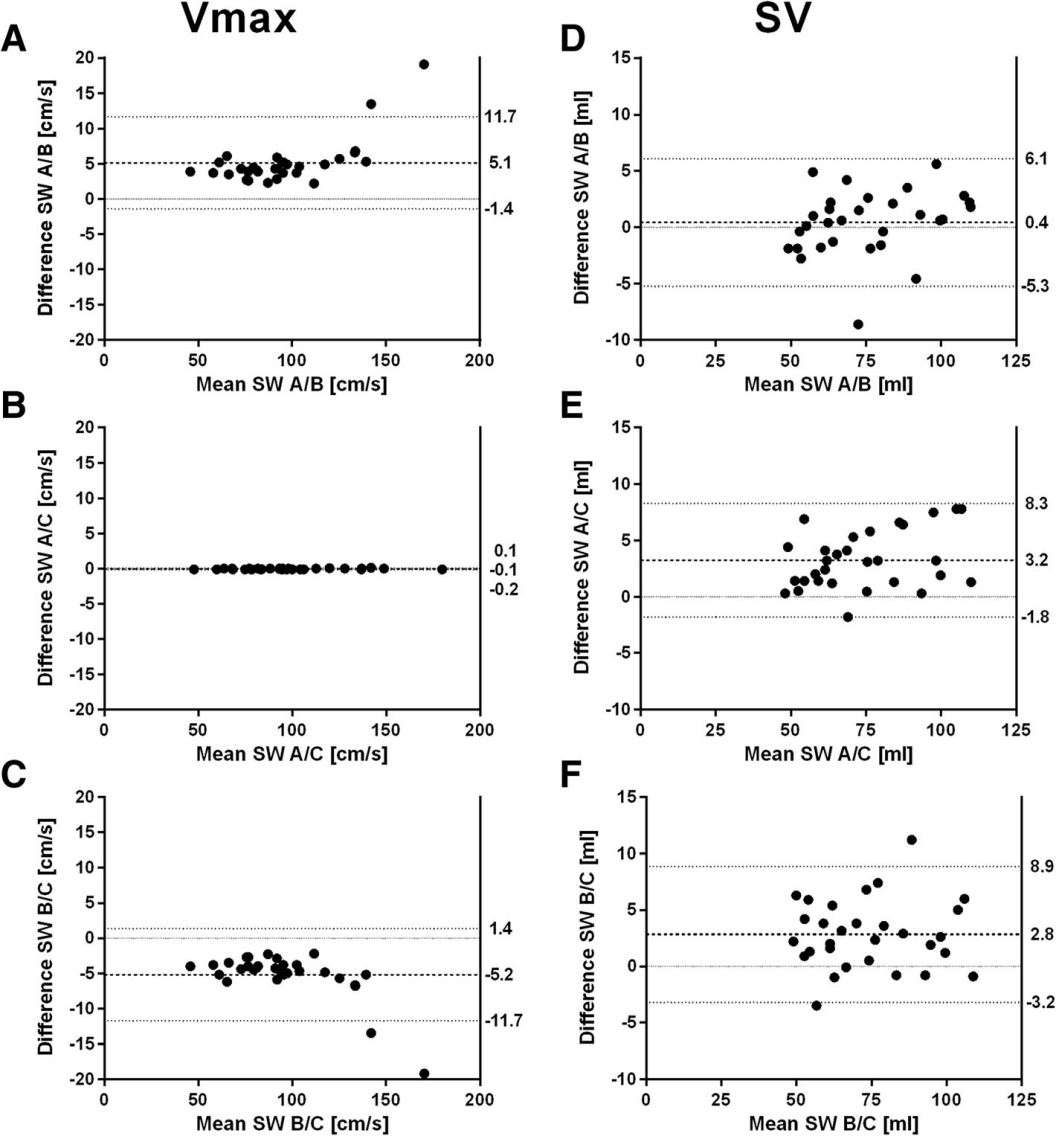
Bland-Altman plots of peak velocity (Vmax) and stroke volume (SV) for agreement between software A and B (**a**, **d**), software A and C (**b**, **e**) and software B and C (**c**, **f**). Dashed lines indicate mean difference, dotted lines indicate limits of agreement

All software showed a very strong positive correlation for Vmax (r: software A/B: 0.996, software A/C: 1.0, software B/C 0.996) and SV (r: S software A/B: 0.989, software A/C: 0.992, software B/C 0.986). For Vmax, bias of software A/C was close to zero and presented narrowest limits of agreement (Fig. 5b). Software B showed lower Vmax compared to software A and software C (Fig. 5a, c). For SV, smallest bias was found between software A/B (Fig. 5d). Software C showed lower SV compared to software A and software B (Fig. 5e, f).

### Parametric mapping

**Fig. 6:**
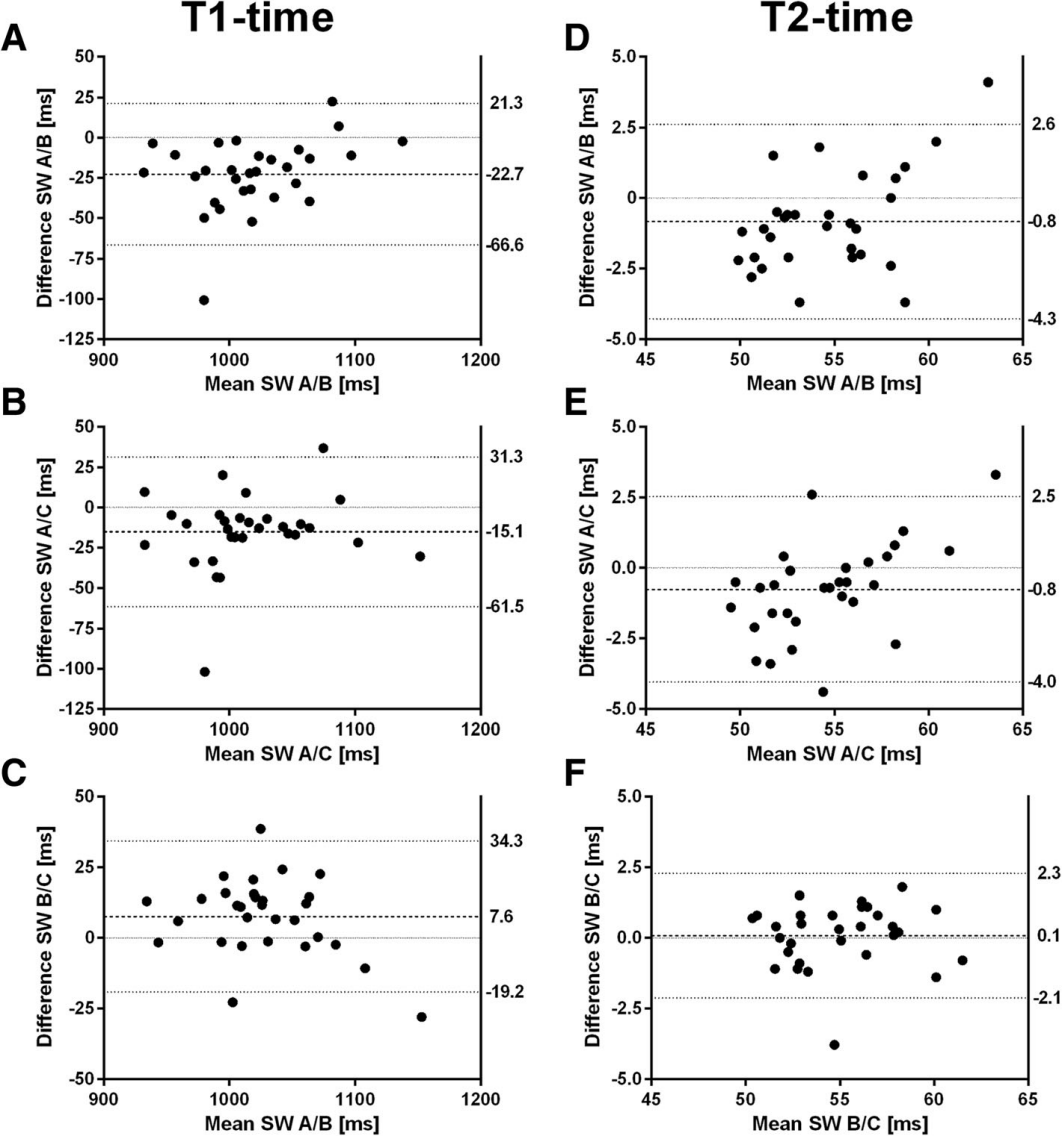
Bland-Altman plots of T1-time and T2-time for agreement between software A and B (**a**, **d**), software A and C (**b**, **e**) and software B and C (**c**, **f**). Dashed lines indicate mean difference, dotted lines indicate limits of agreement

Software B/C showed highest correlation for T1-time (r: S software W A/B: 0.903, software A/C: 0.891, software B/C 0.961) and for T2-time (r: software A/B: 0.897, software A/C 0.912, software B/C 0.931). Software A had longer T1- and T2-time and best agreement was detected between software B/C (Fig. 6a-f). The measured area was significantly smaller in software A compared to software B for both, T1- and T2-time (*p* < 0.001, respectively). Within one software, the measured area did not differ between first and second measurement of T1- and T2-time (*p* > 0.05, respectively).

### Equivalence testing

**Fig. 7:**
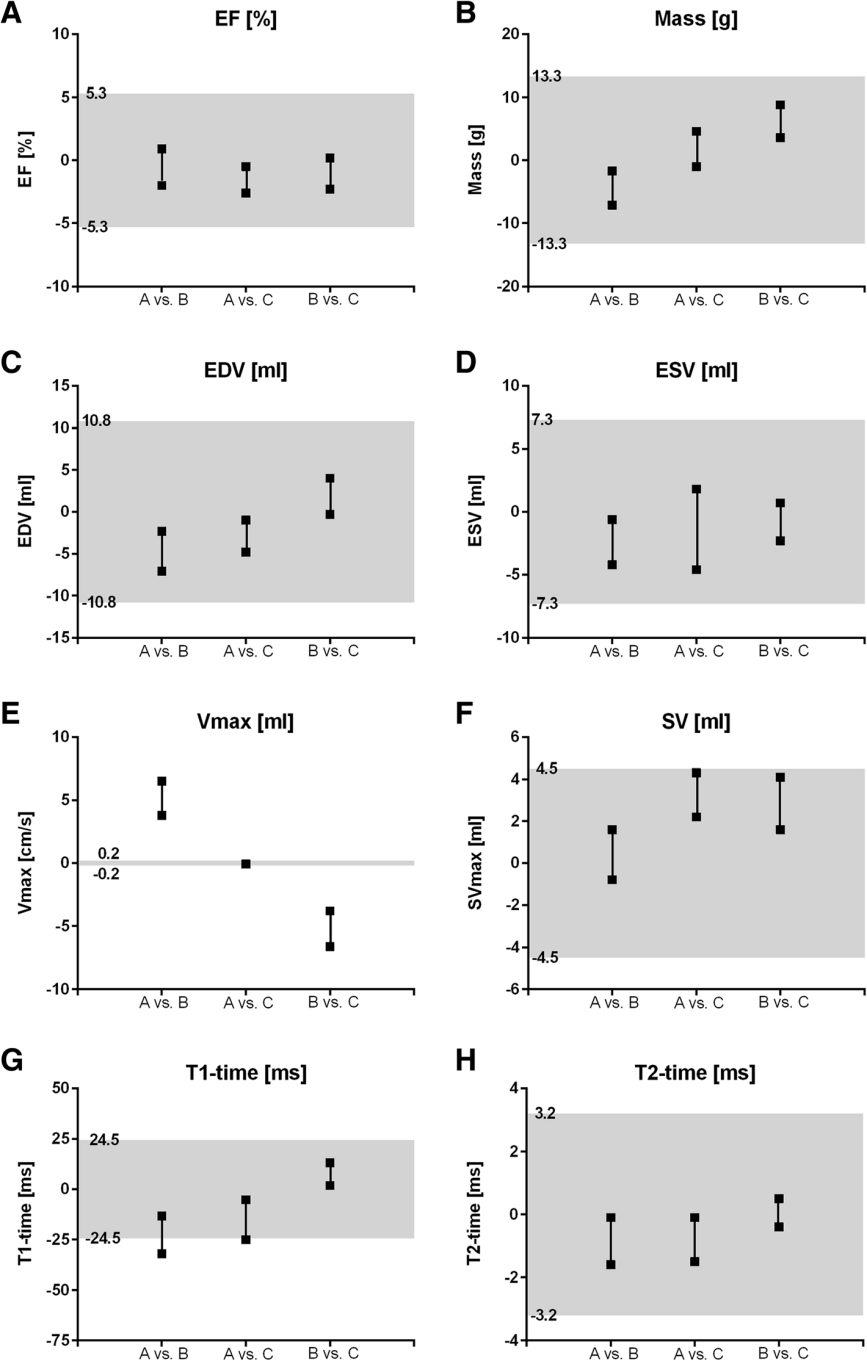
Equivalence testing for LV assessment (**a**-**d**), flow measurement (**e**, **f**), parametric mapping (**g**, **h**). Equivalence of measurements of two software is shown if the confidence interval for software comparison (indicated as black lines, squares marked upper and lower limits) are contained within the equivalence limits (tolerance interval marked grey)

Equivalence limits for the differences between software for each parameter were based on the highest SD obtained by intraobserver analysis and were derived as ±1.96 SD (Table 3, Additional files 1 and 2). Software B showed the highest SD for all parameters except for mass and ESV (software A). For EF, mass, EDV, ESV, SV and T2-time, the Bonferroni-corrected confidence intervals (indicated as black lines in Fig. 7) of all software comparisons were completely contained within the equivalence limits (indicated as grey shaded area in Fig. 7), indicating that software A, B and C could be considered to be equivalent for these parameters (Fig. 7a, b, d, f). For Vmax, software A/C (CI -0.1 to 0.0) were equivalent, (Fig. 7c). In contrast to that, the confidence intervals of the comparisons of software A/B (CI 3.8 to 6.5) and B/C (CI -6.6 to − 3.8) were completely outside of equivalence limits (− 0.2 to 0.2), indicating no equivalence between software A and B as well as between software B and C. For T1-time, equivalence was given between software B and C (CI 1.9 to 13.2) as illustrated in Fig. 7e. The lower confidence intervals of comparisons of software A/B (CI -32.0 to − 13.3) and A/C (CI -25.0 to − 5.3) were marginally outside of the equivalence limits (CI-24.5 to 24.5) signifying that there was not sufficient evidence to claim equivalence.

**Table 3:** Intraobserver variability for software A, B and C

	Software A	Software B	Software C
Left ventricular assessment			
Ejection Fraction [%]	−0.2 ± 2.4	−0.5 **± 2.7**	0.5 ± 1.6
Mass [g]	2.9 **± 6.8**	− 3.4 ± 5.0	− 1.3 ± 3.4
End-diastolic volume [ml]	1.9 ± 5.0	0.6 **± 5.5**	− 0.1 ± 3.4
End-systolic volume [ml]	0.6 **± 3.7**	0.6 ± 2.9	0.3 ± 2.3
2D Flow measurement			
Vmax [cm/s]	0.0 ± 0.0	0.0 **± 0.1**	0.0 ± 0.0
Stroke Volume [ml]	−1.0 ± 2.1	−1.2 **± 2.3**	0.0 ± 1.6
Parametric mapping			
T1-time [ms]	−9.4 ± 9.0	−4.0 **± 12.5**	−0.6 ± 12.2
T2-time [ms]	− 0.4 ± 0.9	−0.1 **± 1.6**	−0.1 ± 1.1

Intraobserver variability expressed as mean bias ± standard deviation. The greatest standard deviation among the three software is indicated in bold

*EF* ejection fraction, *EDV* end-diastolic volume, *ESV* end-systolic volume, *Vmax* peak velocity, *SV* stroke volume

## Discussion

Quantification is a basic requirement for cardiovascular decision making and several parameters in CMR depend on reliable and robust values. To the best of our knowledge, this is the first study comparing three CMR analysis software for quantification of LV 2D flow and T1 and T2 parametric mapping. Main findings were: (i) all three software were equivalent for LV assessment (EF, EDV, ESV and mass), (ii) all three software were equivalent for SV, but only two software for Vmax, (iii) equivalence was given for all software in quantification of T2-time, but only two software for T1-time.

It is well known that different post-processing SW are used world-wide in clinical routine and research. They differ e.g. regarding pixel definition settings, contour detection and other algorithms. Each pixel of a cardiac image displayed by the post-processing software provides information about its size and specific value, such as maximum velocity in case of flow measurement or T1-time in case of T1-mapping. For quantitative image analysis contours intersect pixels. Depending on the software type, different pixel inclusion methods for calculations can be used, e.g. to involve the pixel partly or entirely. In a clinical setting it is crucial to know if these potential differences could impact the results. Previous studies compared the relation between software using correlations, intra-class-coefficient and significant differences. We applied an equivalence testing approach using the intraobserver variability to define equivalence margins to identify deviations between software. In the present study there is no impact of scan procedure related technical influences [21] as we analyzed the same data sets with all three software. The discussion of the results is based on the findings of the particular software versions, we have used. All vendors were open-minded for discussion and adaption.

For LV assessment, all three software showed a high correlation and equivalence for LV EF, EDV, ESV and mass. Our results are supported by previous studies using different software. Messali et al. revealed a high correlation of LV function and volume without significant differences between ViewForum (Philips) and Argus in 46 patients [19]. Kara et al. demonstrated a high correlation between LV tutorials (Cardiovascular Imaging Solutions) and Argus in 40 patients with known or suspected coronary artery disease. Additionally, they compared CMR software with other modalities like CT and 2D echocardiography, but only for EDV they could show a stronger correlation between CMR tools and CT rather than the two CMR software. Another group compared image analysis of 15 healthy subjects between one scanner providing MASS and one scanner providing Argus and did not find significant differences within one observer [41]. Nevertheless, CMR image segmentation is reader dependent and LV quantification differs even between expert readers which emphasizes the need for standardization [42]. In our study, we assumed that a range within software could be declared as equivalent, however, this range would depend on the reader’s precision. Still, our intraobserver bias was comparable to former results even though we excluded papillary muscles from LV mass [36, 43]. In the present study, each software calculated volumes in function of area and slice thickness. As there was no gap between the SAX slices, interpolation was not necessary. EF and mass were derived from cardiac volumes. We conclude that different pixel definitions of the present software did not substantially influence results of LV volumetry. The applied software are interchangeable for LV assessment in this cohort of patients.

Hemodynamics can be assessed by PC-CMR to evaluate shunt fraction, valve regurgitation or stenosis [3]. We used automatic contour propagation with manual correction in all three software for comparison of flow data sets of 30 patients. Boye et al. applied a software flow analysis procedure in 6 patients with aortic insufficiency and showed similar results for aortic regurgitant fraction based on backward/forward SV in four software, three out of those four were the same as in our study [20]. Consistently, the present study showed equivalence for SV between all three software. However, even in phantom measurements without manual contour correction they revealed differences in contour propagation algorithms as they found different velocities among software. In our study, intraobserver analysis of Vmax showed a high reliability within each software. But, despite accurate corrected anatomical borders, we identified software B measuring nonequivalent Vmax values compared to other software even when the peak velocity measuring square was in the same phase and visually at a similar location within the vessel. This finding is attributed to different voxel averaging methods, depending on the software. In software B the default of flow measurement was an averaging including 4 adjacent voxels in contrast to the other software which preset a single voxel. Voxel averaging techniques reduce spatial resolution of the measurement and significantly underestimate peak velocity compared to the single voxel technique with a difference of 7% mean percentage, but do not influence the flow volume [44]. We found nearly congruent Vmax values between software A and C, whereas these software showed the highest bias in SV. This could be explained by the fact that Vmax is measured by only one or a few voxel while SV is calculated as sum of velocities of the voxel within the ROI multiplied by the area at each temporal phase [45]. We cannot exclude small differences in ROI sizes despite manual border correction among software. However, ROI size should then substantially affect the SV which was not the case in this study. Interestingly, the velocity measuring pixel among two software vendors partly exceeded the anatomical and delineated border of the aorta, in turn possibly inducing an incorrect velocity value for this phase. Therefore, attentive care must be taken to control outliers and to avoid misalignment. Other authors analyzed also the impact of different modalities to assess different anatomical structures [46–49]. In our opinion, the validation of different software is warranted at least within an imaging modality and needs further attention.

CMR enables tissue characterization using parametric mapping techniques. Myocardial T2-mapping can detect edema in acute myocardial infarction or inflammation [37]. Native T1-mapping reflects pathological changes in both myocardium and interstitium [35, 50]. It allows further differentiation of cardiac diseases in LV hypertrophy and in systemic diseases such as amyloidosis [51, 52]. For T2* analysis, statistically significant but clinically negligible differences were found between the software Functool protocol (GE) and the T2* module of Qmass [17]. In line with this finding, our results indicated that the present software are not equivalent in quantifying T1-times. Differences could occur due to different contour drawing procedures and pixel inclusion approaches that potentially influence precision. This may lead to the significant smaller area of the ROI in software A than in software B for both, T1- and T2-quantification. Qmass and cvi^42^ provided a tool for endo- and epicardial border delineation. Argus has no such specific tool yet. However, within one software, the delineated area was consistent between two measurements. Another explanation for discrepancies might be the different ranges of the values for T1- and T2-time. This is supported by the fact that the relation of our maximum intraobserver SD to the recently published segment based normal values of our group was much smaller for T1- than for T2-time accounting for narrower equivalence limits for T1-time (the maximum intraobserver SD of ±24.4 ms correlates to ±2.5% of the published normal value of 980.7 ms for T1-time, whereas the maximum intraobserver SD ±3.2 ms correlates to ±6.1% of the published normal value of 52.3 ms for T2-time) [4]. Within one software, SD of intraobserver analysis for T2-time was comparable to other studies using Qmass and Osirix [37, 53]. The intraobserver SD of T1-time is in good agreement with other publications in the literature investigating ViewForum and cvi^42^ [4, 10, 11, 54]. However, the range of published intraobserver values is considerably high. Depending on the CMR sequence a correction factor can be introduced if T1-times have to be calculated using the software [55]. Therefore, the impact of software on T1-time quantification should be evaluated in further studies including other diseases like amyloid and hypertrophic cardiomyopathy and at different sites with an approach to correct for some variations as described for LV assessment [42].

### Limitations

Currently there is a lack of an internationally accepted gold standard for software, like phantoms for the different cardiac structure and function. Therefore, we used intraobserver variability of an experienced reader as gold standard to assess equivalence testing. We investigated only a certain number of SW, being aware that there are many others on the market. Further, our findings were specific for the particular software version, knowing that software packages evolve continuously. We did not analyze different cardiovascular diseases but among the selected patients 52% suffered from cardiac alterations. The potential influence of multiple observers and other pathologies on the comparability of results from different software systems was not considered in this study but should be subject to continuative analyses.

## Conclusion

We could demonstrate exchangeability of cvi^42^ version 5.6, Classic Argus and Medis Suite 2.1 for LV evaluation, forward stroke volume in 2D flow measurement and T2-time in T2 parametric mapping. We conclude that different pixel inclusion methods of the software do not substantially affect calculation of the mentioned parameters but might influence results of T1-time. Vessel contours and the peak velocity measuring square in each phase of flow measurement should be checked carefully, particularly when contour propagation is used. Software users should be aware of the current setting of voxel averaging techniques during flow analysis. Our results underline the need of standardization and indicate that the individual analysis software (including version) and specific settings should be mentioned in clinical reports to avoid misinterpretation upon follow-up examinations and to assure comparability of CMR studies.

## Additional files

Additional file 1: Intraobserver analysis. Bland-Altman plots of LV function (EF), mass, end-diastolic (EDV) and end-systolic volume (ESV) (PDF 369 kb)

Additional file 2: Intraobserver analysis. Bland-Altman plots of peak velocity (Vmax), stroke volume (SV), T1-time and T2-time (PDF 368 kb)

## Abbreviations

bSSFP: Balanced steadystate free precession
CMR: Cardiovascular magnetic resonance
ECG: Electrocardiogram
EDV: End-diastolic volume
EF: Ejection fraction
ESV: End-systolic volume
LV: Left ventricle/left ventricular
PC: Phase contrast
ROI: Region of interest
SAX: Short axis
SD: Standard deviation
SV: Stroke volume
Vmax: Maximum velocity, peak velocity
