# Supplementary figures and images for: Quantification in cardiovascular magnetic resonance: agreement of software from three different vendors on assessment of left ventricular function, 2D flow and parametric mapping
Source: J Cardiovasc Magn Reson. 2019 Feb 21;21:12. doi: 10.1186/s12968-019-0522-y (PMC6383230; doi:10.1186/s12968-019-0522-y)

**EF**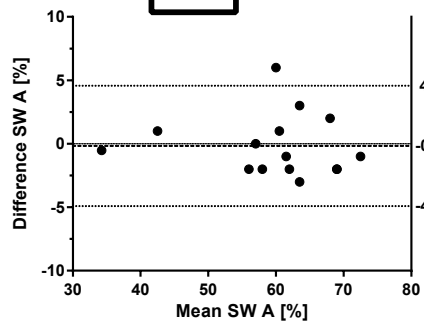**Mass**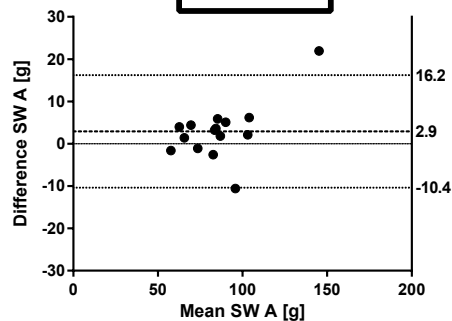**ESV**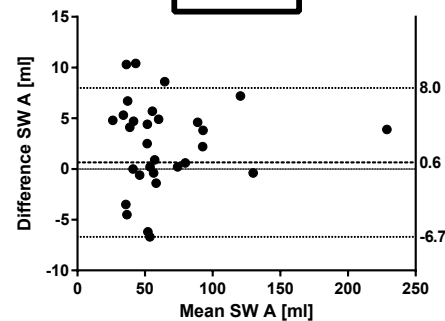**EDV**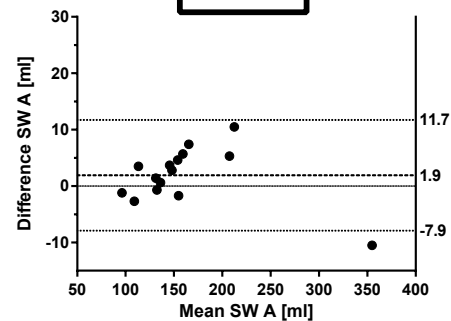**SW B**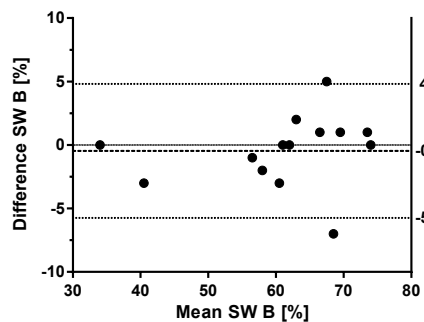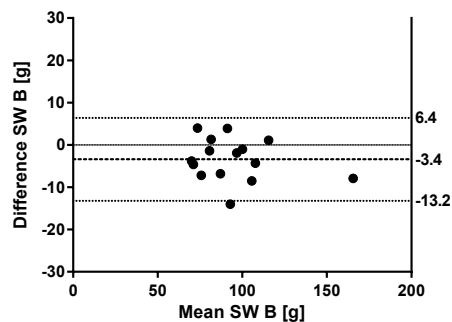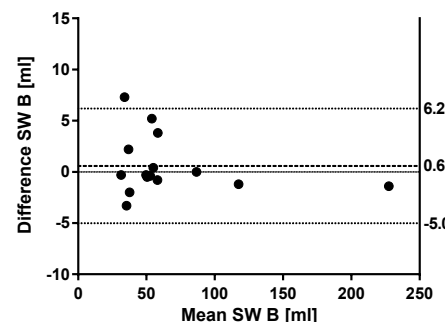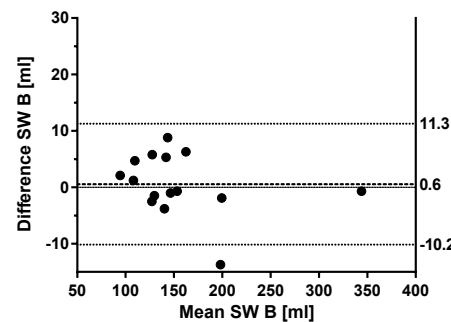**SW C**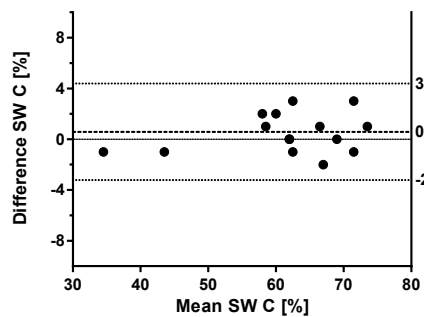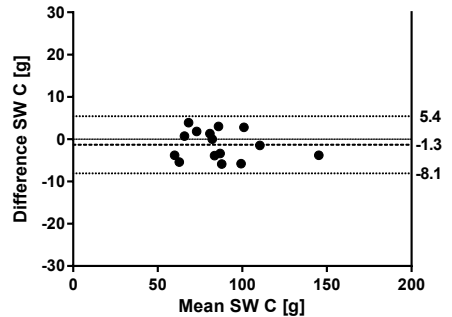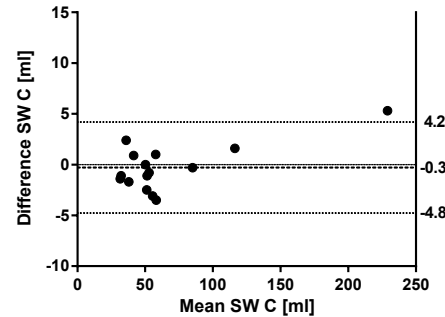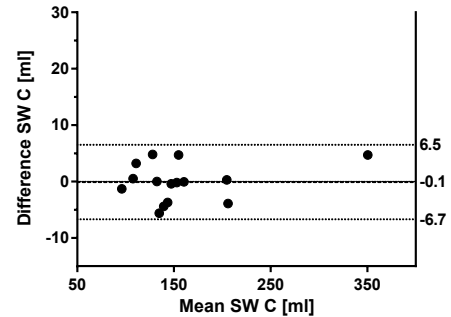

Supplement: Supplementary file 1 — Intraobserver analysis. Bland-Altman plots of LV function (EF), mass, end-diastolic (EDV) and end-systolic volume (ESV) (PDF 369 kb) [file 12968_2019_522_MOESM1_ESM.pdf]

Vmax

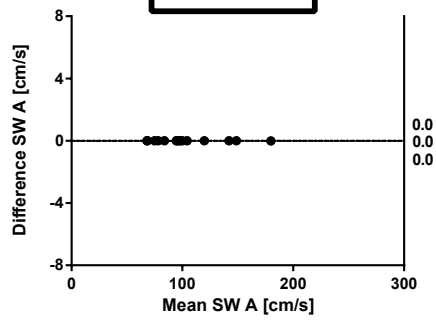

SVmax

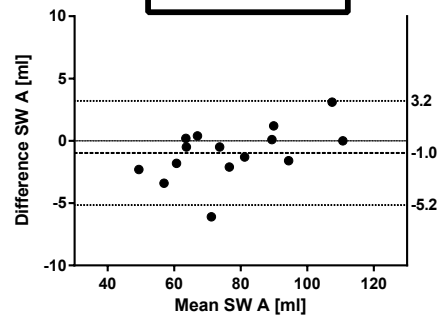

T1

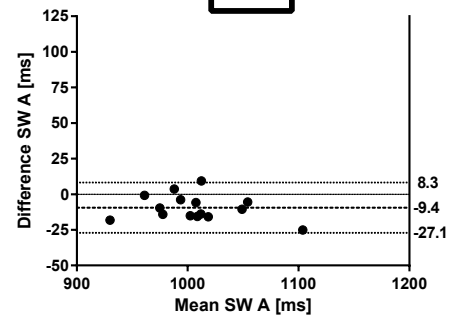

T2

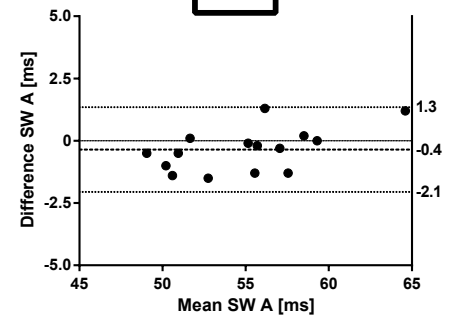

SW A

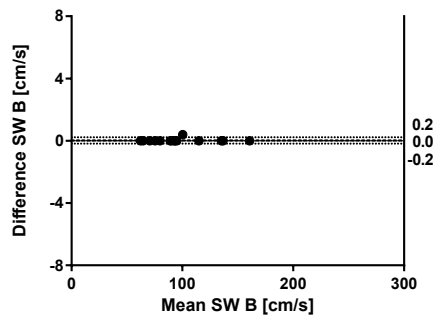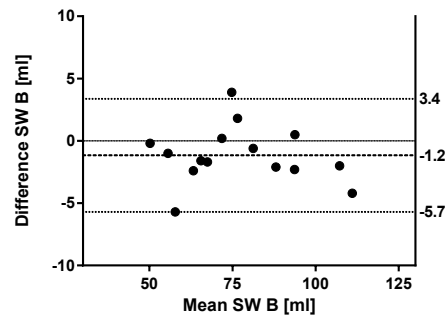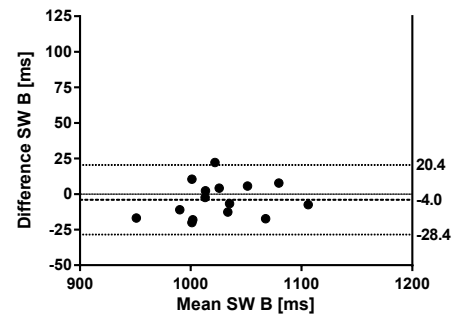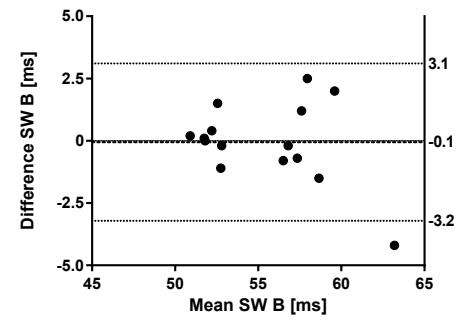

SW B

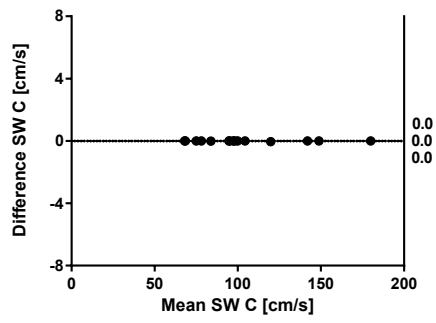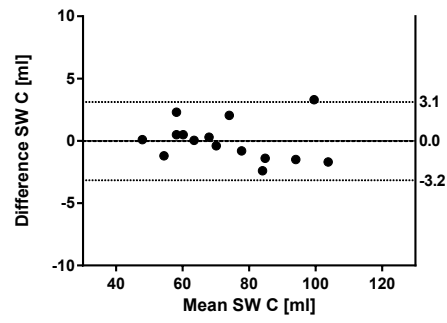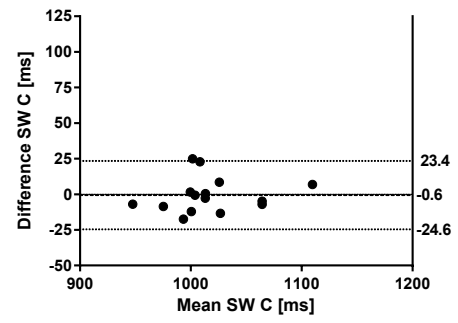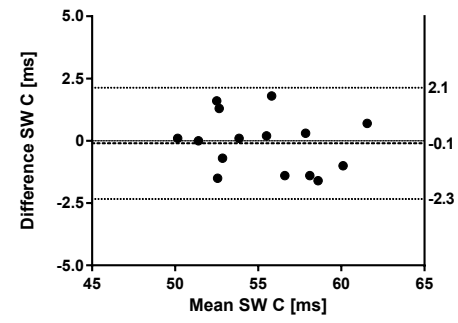

SW C

Supplement: Supplementary file 2 — Intraobserver analysis. Bland-Altman plots of peak velocity (Vmax), stroke volume (SV), T1-time and T2-time (PDF 368 kb) [file 12968_2019_522_MOESM2_ESM.pdf]
